# A Biologically-Inspired Framework for Contour Detection Using Superpixel-Based Candidates and Hierarchical Visual Cues

**DOI:** 10.3390/s151026654

**Published:** 2015-10-20

**Authors:** Xiao Sun, Ke Shang, Delie Ming, Jinwen Tian, Jiayi Ma

**Affiliations:** 1 School of Automation, Huazhong University of Science & Technology, 1037 Luoyu Road, Wuhan 430074, China; E-Mails: sxiao@hust.edu.cn (X.S.); shangke1988@hust.edu.cn (K.S.); jwtian@hust.edu.cn (J.T.); 2 Electronic Information School, Wuhan University, 299 Bayi Road, Wuhan 430072, China; E-Mail: jyma2010@gmail.com

**Keywords:** contour detection, biologically inspired, candidate set, hierarchical visual cues, Gestalt principles

## Abstract

Contour detection has been extensively investigated as a fundamental problem in computer vision. In this study, a biologically-inspired candidate weighting framework is proposed for the challenging task of detecting meaningful contours. In contrast to previous models that detect contours from pixels, a modified superpixel generation processing is proposed to generate a contour candidate set and then weigh the candidates by extracting hierarchical visual cues. We extract the low-level visual local cues to weigh the contour intrinsic property and mid-level visual cues on the basis of Gestalt principles for weighting the contour grouping constraint. Experimental results tested on the BSDS benchmark show that the proposed framework exhibits promising performances to capture meaningful contours in complex scenes.

## 1. Introduction

Contour detection has remained a fundamental problem in computer vision that has been intensively studied in the past fifty years. It is a crucial step for many computer vision tasks, including object segmentation [1,2], shape-based object matching [3,4,5,6], detection and recognition [7,8,9,10,11,12,13], image restoration [14,15,16], recovery of intrinsic scene properties [17], *etc*. Unlike edges, which refer to any abrupt change in local image features, such as luminance and color, contour represents changes from object to background or one surface to another, which need deeper levels of image information to detect.

A large number of methods have been proposed for edge or contour detection in recent decades. Typical methods include local linear filters [18,19,20,21], active contours [22,23,24], learning or statistical inference methods [25,26,27,28,29,30,31]. Due to the broad diversity and ambiguity of visual patterns in a natural image, it remains a challenging task to automatically extract image contours with a computer accurately and efficiently, even though much progress has been made.

However, the human vision system is able to acquire relative integration and structure from images without any prior knowledge of objects. Research has shown that the brain does not directly obtain the projected images on the retina presented by the external stimuli. Instead, it recognizes objects from visual information that has been aggregated and specialized [32]. Neurons in lateral geniculate nucleus (LGN) and primary visual cortex (V1 or striate cortex) respond to oriented luminance and chromatic stimulation and extract relevant information from natural images that have specific characteristics through this low-level processing. Additionally, the main function of the later visual cortex is to perform the extraction and calculation of perceptual signals, in a way that dramatically reduces the data volume, but retains the useful structural information of the objects. In a complex natural scene, the components that have abundant and specific attributes are more prominent and are thus easier to isolate from the background. This problem of extracting and integrating structural information is called perceptual grouping. The process of perceptual grouping is often considered as being guided by the middle-level Gestalt principle from the psychological perspective. In the Gestalt principle, the visual system tends to group consistent image features into high-level structures through some rules, including brightness and contrast similarity, good continuation, local proximity and curvature constancy. A series of neurophysiological supporting evidence of these properties has been studied in [33,34,35,36] (see [37] for a review). Inspired by these biological bases, many works for contour detection have been developed, for example [38,39,40,41,42,43,44,45,46,47,48].

Almost all of these existing contour detection methods, either biologically-inspired models or computational ones, are conducted on discrete pixels. However, discrete pixel-based methods extract features of each pixel in its local patch regardless of whether or not similar pixels need to be computed repeatedly. They often have a large computational cost and are more likely to generate discontinuous or broken contours than the methods conducted in larger primitives, especially when suffering from noises. Larger primitive conducting and a smaller search space are new trends of image processing or vision tasks. Therefore, superpixel-based methods and candidate-based frameworks have become hotspots in tasks such as object segmentation [49] or object detection [50] in recent years. However, there are few contour detection algorithms based on these methods and frameworks.

In our work, we propose a biologically-inspired candidate weighting framework for contour detection. To generate contour candidates for reducing the computation space, we introduce a modified superpixel generation processing. Our basic assumption is that the contours of the meaningful objects align well with superpixel boundaries. Based on that, the superpixel boundaries can be taken as contour candidates after segmenting the input image into superpixels. Therefore, the problem of detecting contours is transformed into another one, weighing or estimating the probability of being an object contour of each candidate. Then, we propose a biologically-inspired method of extracting hierarchical visual cues to weight the candidates. The lower level of this hierarchical visual system responds to oriented luminance and chromatic stimulation and is thus used to weight intrinsic properties of contours. The mid-level of the hierarchical visual system groups perceptual signals by Gestalt principles, resulting in the contour connection constraint. Our proposed method has been tested on the BSDS 300/500 benchmarks [1,26], and experimental results exhibit promising performances.

The remainder of this paper is organized as follows. In Section 2, we present related work in contour detection and the candidate-based framework. Then, we describe the details of our candidate-based contour detection framework and biologically-inspired candidate weighting algorithm from hierarchical visual cues in Section 3. In Section 4, we evaluate the performance of the proposed method on the BSDS 300 and 500 datasets [1,26]. Finally, we discuss our model and draw conclusions in Section 5.

## 2. Related Work

Contour detection and edge detection are classical problems in computer vision. There is an enormous literature on these topics. It is hard for this paper to give a full survey on the topic, so only a small relevant subset of works will be reviewed here.

The early approaches to contour detection relied on local measurements with linear filters. Typical algorithms are Sobel [18], Robert [19], Prewitt [20] and Canny [21] edge detectors. All of these method use local filters of a fixed scale and few orientations. Small and unimportant edges are treated equally as meaningful contours in these detectors. This will lead to noisy contour maps, which are hard to use for subsequent higher level processing. The key challenge is to enhance the weights of stable and meaningful contours without enhancing gradients due to repeated or stochastic textures.

Therefore, over the years, many new approaches have been developed for contour detection. Typically, Malik *et al*. [1,26] defined gradient operators for multiple cues, such as brightness, color and texture. A regression classifier is used to predict edge strength from these features. This is the popular *Pb* algorithm [26], which learns to give each pixel in the image the probability of a contour at that point. Then, multiple scales [51] and multiple features [52] have been incorporated into learning-based contour detection algorithms. In the last five years, other learning methods were proposed. Ren and Bo [30] used sparse coding and oriented gradients to learn dictionaries of contour patches. Lim *et al*. [28] used random forest-based learning on image patches and achieved state-of-the-art results. Their key idea is to use a dictionary of human-generated contours as features for contours within a patch. Dollar and Zitnick [29] further combined random forests with structured prediction to achieve real-time edge detection. However, the performances of most learning-based methods are strongly dependent on the selection of training sets, which makes the methods inflexible for an individual image. Furthermore, training a method on a dataset normally leads to a high computational cost.

Besides, many non-learning-based algorithms have been proposed for boundary detection recently. The research was focused on a global framework that minimized the global cost over all disjoint pairs of patches. For example, Felzenszwalb and McAllester [53] extracted salient contours by solving the min-cover problem. Arbelaez *et al*. [1] embedded the local *Pb* measure [26] into a spectral clustering framework. Isola *et al*. [54] measured rarity based on pointwise mutual information. They also obtained good results, although the computation cost is exhausted.

Another line is the success of biologically-inspired methods. From neuro-physiological perspectives, it has been widely believed that the neurons in V1 are exquisitely sensitive to oriented bars or edges in the classical receptive field (CRF). Subsequently, extensive neuro-physiological findings indicate that a peripheral region beyond CRF, known as non-classical receptive field (non-CRF), can modulate the spiking response of a V1 neuron to the stimuli placed within the CRF. The neuronal responses are strongly inhibited when the stimuli within the CRF and non-CRF share similar features. Based on this physiological mechanism, several biologically-inspired contour detection methods have been proposed recently [38,39,40,41,42,43,44,45,46,47,55]. These methods modeled the orientation-selective excitation mechanism of CRF with orientated Gabor or derivate Gaussian filters, with rare exceptions. For surroundings inhibition, many studies proposed different models, like [32,47,55,56,57], and used another strategy of the visual system to cope with complex scenes in the last five years. For instance, multiple scales [32] and multiple visual features [55] were integrated. In addition, an efficient color boundary detection framework was proposed by simulating the biological mechanisms of color information processing and color-opponent mechanisms along the retina-LGN-cortex visual pathway [48]. From the psychophysical perspective, psychologists formulated Gestalt rules for perceptually-significant image structure during contour grouping, such as “proximity”, “good continuity”, “closure”, *etc*. Gestalt-based contour grouping or detection algorithms have also been studied recently. In [58,59], an optimal Bayesian model was employed to investigate the inferential power of three classical Gestalt cues: proximity, good continuation and luminance similarity for contour grouping. Hess *et al*. [37] discussed the mechanism of contour integration from psychophysical and neurophysiological perspectives and summarized some computational models. In [60], a novel higher order CRF model was proposed to address the contour closure effect through local connectedness approximation. Han *et al*. [61] proposed a fuzzy connection facilitation model to achieve the enhancement of contour response and the connection of discontinuous contour. These biologically-inspired methods also achieved good results.

The above methods commonly use pixel-level measurements to create contours. However, these methods often lead to noisy and broken contours, which are costly and less likely to be useful for further processing. Superpixels provide a convenient primitive and have become increasingly popular for use in computer vision applications. A series of superpixel generating algorithms have been proposed. One major category is graph-based approaches, like [62,63,64,65]. These approaches treat each pixel as a node in a graph and use edge weights between two nodes to measure the similarity between neighboring pixels. Superpixels are created by minimizing a cost function defined over the graph. Another major category is gradient ascent-based algorithms [66,67,68,69,70,71]. These kinds of algorithms start from a rough initial clustering of pixels, then use gradient ascent methods iteratively refining the clusters until some convergence criterion is met to form superpixels. In addition, candidate-based frameworks have emerged as a new mechanism to reduce computation costs in vision tasks. For example, objectness-based bounding box candidates have been applied for object detection [50]. Object hypotheses have been represented as figure-ground segmentation proposed in [72] and used as a basis of salient object segmentation [49]. However, to the best of our knowledge, similar algorithms or frameworks have been rarely used to detect contours.

In our work, a biologically-inspired candidate weighting (BICW) framework is proposed for contour detection. We introduce a modified superpixel generation processing to obtain contour candidates and then weigh the candidates by multiple biological cues.

## 3. Method of Biologically-Inspired Candidate Weighting Framework for Contour Detection

**Figure 1:**
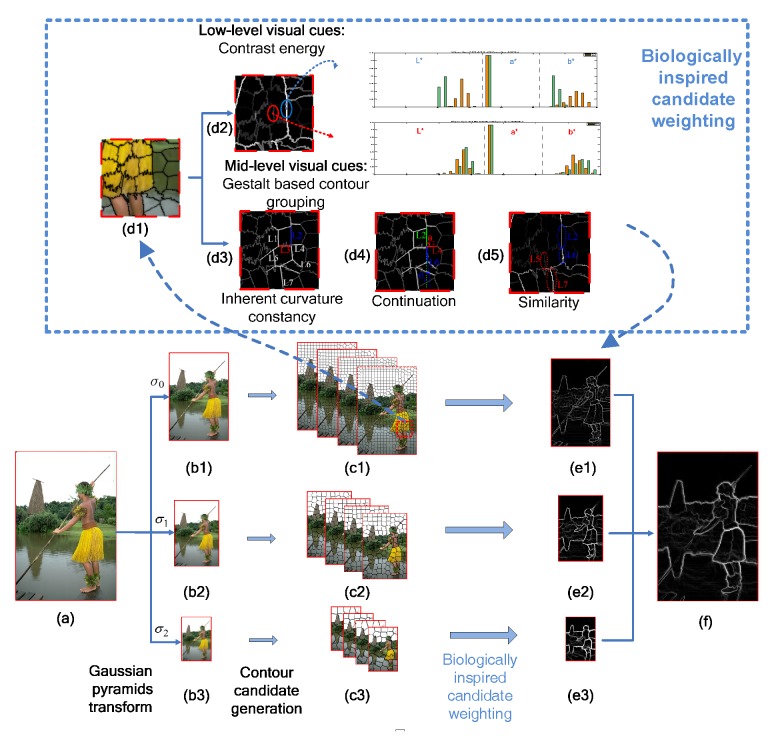
Flowchart of our contour detection framework. (**a**) Input image; (**b1**–**b3**) transformed images of different scales ($\sigma_{0}$, $\sigma_{1}$, $\sigma_{2}$); (**c1**–**c3**) contour candidates generated by our modified simple linear iterative clustering (SLIC) processing. We perform SLIC with four different initials in each scale. To facilitate the expression, we draw the candidates on four images in each scale, which together form the candidate set; (**d1**) Zoomed-in region for the demonstration; (**d2**) contrast energy extracted with low-level cues; (**d3**) inherent curvature constancy extracted with mid-level cues; L2 labeled by the blue line indicates the candidate with good inherent curvature constancy, which is more likely to be a true contour, while L3 labeled by a red line indicates poor inherent curvature constancy; (**d4**) continuation extracted with mid-level cues. A true contour candidate tends to connect to its co-linear neighbor, but is independent from the vertical one. For example, L6 is more likely to link with L2, but L4 is slightly related to L2; (**d5**) Similarity extracted with mid-level cues. Connection-probability attenuates with the increasing of contrast difference, for example L2 and L6 labeled by blue-dotted circles yield a higher connection-probability than L5 and L7 labeled by red-dotted circles; (**e**) Weighted candidates on an image at each scale; (**f**) final output.

In this section, we will describe our contour detection framework. A detailed flowchart is shown in Figure 1. Firstly, in Section 3.1, a modified superpixel generation processing with a multi-scale strategy is introduced to get a set of contour candidates. Then, we propose our biologically-inspired method for weighting the contour candidates from hierarchical visual cues in Section 3.2.

### 3.1. Generation of Contour Candidates

Our first step is to generate contour candidates by the superpixel generation algorithm. A modified simple linear iterative clustering (SLIC) [66] algorithm is used to obtain the superpixels from the input image. Superpixel boundaries are subsequently obtained as contour candidates. The computations of this method are not intended as a model of biological visual processing. However, these computations are intended to provide closed and continuous edges, which seems to be more in line with the Gestalt principles than a biologically-inspired edge detection algorithm that uses a derivative Gaussian filter or classical hand-designed methods, such as a Canny detector.

SLIC is an adaptation of K-means for superpixel generation, which is faster and more memory efficient than existing superpixel generation methods. SLIC also exhibits state-of-the-art boundary adherence. In this method, the *k* cluster centers sampled on a regular grid space with *S* pixels are initialized separately, where *S* is the grid interval and *k* is defined as $K=N/S^{2}$. Each pixel is then iteratively associated with the nearest cluster center measured by the 5D Euclidean distance in the labxy space, whose search region is limited to 2S×2S around the center.

If the assumption that the contour of the meaningful objects aligns well with superpixel boundaries is valid, in other words, if the boundary recall is sufficiently high, superpixel boundaries can be taken as contour candidates. The boundary recall of SLIC has been discussed in [66]. Its experiment showed that the boundary recall increased with the number of superpixels, and SLIC demonstrated an optimum performance. We can see from the SLIC algorithm calculation process shown above that the only parameter of SLIC is the desired number of approximately equally-sized superpixels *k*, which determines the initial clustering centers in the initialization step and the cluster searching scope in the assignment step. In our work, we make a modification of the original SLIC processing. Instead of changing *k* of the original SLIC to get better performance, we separately process SLIC with different initial cluster centers. We change the initial cluster center with some offsets and generate a series of superpixels sp with different initial centers. Therefore, the contour candidate set at the $\sigma_{j}$ scale can be obtained as follows:

(1) $$B_{\sigma_{j}}=\left\{B_{i,\sigma_{j}}=boundaries\;of\;superpixel\;i_{1}\;and\;i_{2}\;|\;i_{1},i_{2}\in sp_{\sigma_{j}},i_{1}\neq i_{2}\right\}$$

We experimentally validated the completeness of the candidate set generated from this method, which will be explained in detail later in Section 4. Therefore, contour detection can be simplified as another problem of weighting candidates with the probability of being the object contour from hierarchical visual cues.

**Figure 2:**
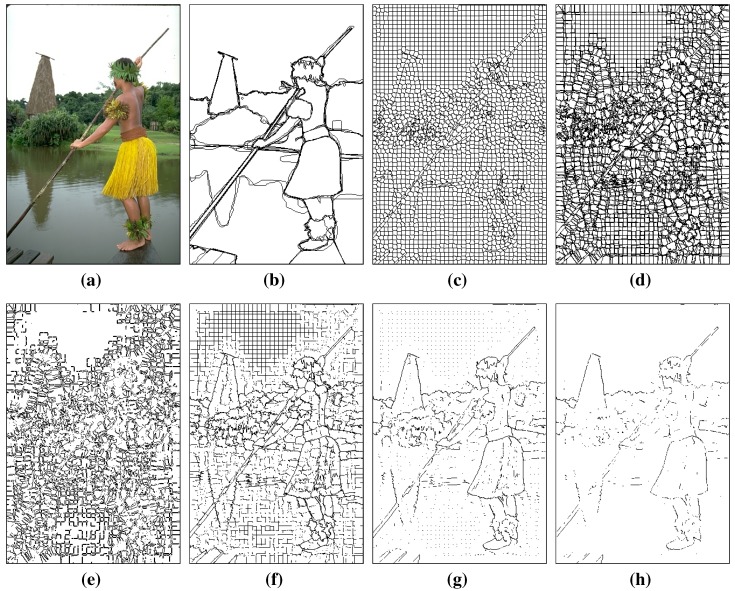
Contour candidates generated by original SLIC and our modified SLIC. (**a**) Input image; (**b**) ground truth; (**c**) contour candidates generated by original SLIC; (**d**) contour candidates generated by our modified SLIC, superimposed on all of the contour candidates over this image; (**e**) Pixels appear only once in contour candidates; many of them are clutter or meaningless textures; (**f**)–(**h**) Pixels appear more than once, twice and three times; pixels of meaningful contour are preserved to be candidates with different initials.

The advantage of our modified superpixel generation method is that the weight of stable edges, which are more likely to be a contour, can be increased. In Figure 2, we can see that stable edges are still the boundaries of superpixels when the initial cluster centers are different. By contrast, the unstable edges are easily altered. In our method, the pixels of stable edges occur multiple times as different candidates. When we sum the weights of each candidate to get the final probability of being a true contour, the pixels of the stable edges yield higher weights than those of the unstable edges.

The contour candidates are derived from superpixel boundaries, which are formed by the adjacent superpixels. Thus, we only need to extract features of each contour candidate from adjacent superpixels. In previous work, the features of each pixel are extracted in its local patch. Similar pixels in its neighborhood will be computed repeatedly. Compared to the previous approach, our method can reduce many repeated calculations brought by these similar pixels and weaken the influence of noises, because pixels are grouped into perceptually-meaningful atomic regions by using our superpixel-based candidate generation method.

The information obtained at different scales is different, so the resulting contours are also different. At a small scale, information, including contours and some textures, is mostly retained. The main contours of the target object are retained at a larger scale, but some details may be missing. Salient and meaningful contours are more likely to be retained at different scales, which is called continuity at scales. To handle the scale problem and to use the continuity property to obtain more meaningful contours, we generate contour candidates and weigh them at s+1 different scales. In previous work, the convolution kernel [32,51] or measure scope [1] is changed to obtain images of multiple scales, but the image size is unchanged. Different from previous work, we obtain the transforming image of different scales through Gaussian pyramid down-sampling transformation on account of the time and space cost. The modified candidate generation operation is then performed on transformed images of each scale. The total contour candidate set is expressed as follows:

(2) $$B=B_{\sigma_{0}}\cup B_{\sigma_{1}}\cup \cdots \cup B_{\sigma_{s}}$$

In this work, we consider weights at three scales (s=2), where $\sigma_{1}=2\sigma_{0}$ and $\sigma_{2}=4\sigma_{0}$. Here, only integer multiple scales are considered to avoid interpolation during Gaussian pyramid down-sampling transformation.

### 3.2. Extraction of Hierarchical Cues

The human visual system has evolved to extract relevant information hierarchically from natural images with specific characteristics. First, neurons in primary visual cortex (V1 or striate cortex) respond to oriented luminance and chromatic stimulation, which is expressed as a low-level visual process. Then, the mid-level process performs some kind of grouping operation in both V1 and later visual areas. This process provides a bridge between low-level local information and high-level concepts, such as object- and scene-level information. The rules of contour grouping are also formulated by the Gestalt “law” in the psychological perspective [37]. In this work, we propose a hierarchical cue extraction method that extracts low-level visual local cues to weigh contour intrinsic properties and mid-level visual cues based on Gestalt principles to weigh the contour grouping constraint, as described in this subsection.

#### 3.2.1. Low-Level Cues

Local luminance and chromatic information are important visual low-level cue features to understand natural scenes. In this work, the contour intrinsic property is weighted with luminance and chromatic contrast. We measure the luminance and chromatic cues from the input color image using the CIELAB color space, which is a good expression for both luminance and chromaticity. For the luminance cue of each superpixel, we compute a histogram of $L^{*}$ values. For the chromatic cue, it presents additional challenges for estimation because the pixel values are in the 2D space ($a^{*}$ and $b^{*}$). The joint approach is far more expensive computationally than the marginal one due to the additional dimension in histograms. One might expect that the joint color distribution using the 2D histogram contains more visual perceptual information than the marginal one. However, their performances do not remarkably differ, since the $a^{*}$ and $b^{*}$ axes in the CIELAB color space are designed to mimic the blue-yellow and green-red color opponency, which is found in the human visual cortex [73]. Following the method in [26], we compute the marginal color values for $a^{*}$ and $b^{*}$, rather than compute the joint ones. We make a histogram of the values of each channel $\left(L^{*},a^{*},b^{*}\right)$ of each superpixel with $N_{L}$, $N_{a}$ and $N_{b}$ bins, respectively:

(3) $$\begin{array}{cc} L & ={\left[L_{1},L_{2},\cdots ,L_{N_{L}}\right]}^{T} \end{array}$$

(4) $$\begin{array}{cc} a & ={\left[a_{1},a_{2},\cdots ,a_{N_{a}}\right]}^{T} \end{array}$$

(5) $$\begin{array}{cc} b & ={\left[b_{1},b_{2},\cdots ,b_{N_{b}}\right]}^{T} \end{array}$$

Here, we set $N_{L}=16$, $N_{a}=8$ and $N_{b}=8$ according to [26].

As shown in Figure 1d2, the histogram vectors between adjacent superpixels on the sides of a meaningful contour (positive candidate) are quite different, while vectors between negative ones are similar. We use these vectors to compute the contrast energy of the contour candidate between superpixels and additional mid-level cues. The contrast energy of the contour candidate $B_{i}$ formed by the adjacent superpixels $i_{1}$ and $i_{2}$ is expressed by the weighted sum of the distances between superpixels $i_{1}$ and $i_{2}$ based on luminance and chromatic cues, described as follows:

(6) $$E\left(B_{i}\right)=E\left(i_{1},i_{2}\right)=\sum_{c}\alpha_{c}d_{c}\left(i_{1},i_{2}\right)$$

where *c* indexes feature channels (luminance $L^{*}$, chromatic $a^{*}$ and $b^{*}$), and $\alpha_{c}$ indicates the relative contribution of each color channel. $d_{c}\left(i_{1},i_{2}\right)$ is the $\chi^{2}$ distance between histograms of superpixel $i_{1}$ and $i_{2}$ in channel *c*.

#### 3.2.2. Mid-Level Cues

For the mid-level visual process, the visual system groups local edge information into contours guided by the Gestalt principles. In the Gestalt principle, the visual system tends to group consistent image features into high-level structures through some rules, including brightness and contrast similarity, good continuation, local proximity and curvature constancy. Thus, on the basis of these rules, we propose a model for weighting the grouping constraint of contour candidates by computing the connection probabilities with the adjacent candidates.

**Figure 3:**
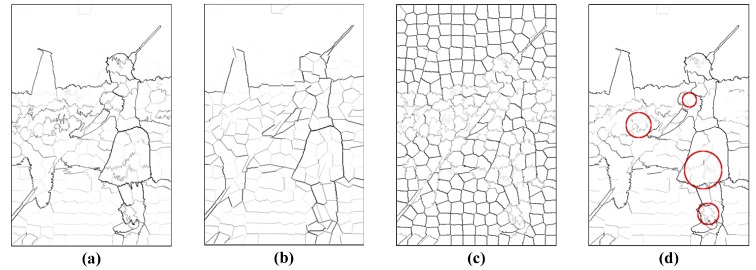
Inherent curvature constancy. (**a**) Contrast energy of the contour candidates computed by low-level cues, using the gray image to represent the contrast energy of each candidate; (**b**) fitted straight line of each candidate with the contrast energy weight; (**c**) inherent curvature constancy evaluation of each candidate; clutter textures tend to be jagged and have small evaluation values; (**d**) modulated weight after inherent curvature constancy evaluation; note that clutter textures marked by red circles have been suppressed obviously compared to (a).

Although our contour candidates yield an accurate representation for the image, these candidates do not provide an optimal basis for contour grouping, as they would be jagged and noisy to some extent. On the other hand, a contour candidate is less likely to be a contour if it is jagged or sensitive to noises, which does not conform to the “curvature constancy” rules. To avoid these problems and to measure the inherent curvature changes of a contour candidate $B_{i}$, we fit it to a straight-line $L_{i}$ and evaluate the inherent curvature constancy of the contour candidate as follows:

(7) $$P_{in-curv}\left(B_{i}\right)=\frac{l_{i}}{b_{i}}$$

where $l_{i}$ is the length of the fitting line $L_{i}$ and $b_{i}$ is the number of pixels of contour candidate $B_{i}$. Figure 3 presents an example of the effect of the inherent constancy constraint.

According to the rules of continuity, a true contour candidate tends to be connected to its neighbors if they are aligned along a linear or co-circular path. In previous work [41,58,59], the co-circularity rule is encoded with the direction of a pixel and its neighboring ones and the angle of the line connecting these pixels. However, the angle of the line connection in our work is redundant and difficult to formulate because continuation and proximity have been concerned to some extent in our contour candidate generation method. Thus, we encode a continuation connection probability with the differences in the direction of the fitting straight lines $L_{i}$ and $L_{j}$ of contour candidates $B_{i}$ and $B_{j}$:

(8) $$P_{cont}\left(B_{i},B_{j}\right)=1-\sin\left({\left|\theta_{i}-\theta_{j}\right|}^{+}\right)$$

where ${\left|\theta_{i}-\theta_{j}\right|}^{+}=\min\left(\left|\theta_{i}-\theta_{j}\right|,\pi -\left|\theta_{i}-\theta_{j}\right|\right)$; $\theta_{i},\theta_{j}$ are the directions of $L_{i}$ and $L_{j}$, respectively. Note that when ${\left|\theta_{i}-\theta_{j}\right|}^{+}$ increases, these two candidates tend to be vertical and have less impact on each other, that is to say the continuation connection probability $P_{c}\left(B_{i},B_{j}\right)$ is small.

For similarity, the connection-probability function should be attenuated with the increasing of contrast difference. Thus, we define the similarity connection probability between contour candidate $B_{i}$ and $B_{j}$ as follows:

(9) $$P_{sim}\left(B_{i},B_{j}\right)=\exp\left(-\frac{d_{contrast}^{2}\left(B_{i},B_{j}\right)}{2\delta^{2}}\right)$$

where the standard deviation δ establishes the sensitivity of the connection probability with contrast difference. Contrast difference and low-level feature difference are calculated using the following equation:

(10) $$d_{contrast}\left(B_{i},B_{j}\right)=E\left(i_{1},i_{2}\right)-E\left(j_{1},j_{2}\right)$$

#### 3.2.3. Combination of Hierarchical Cues

Combined with these elements, the weight of being a contour for candidate $B_{i}$ is defined as the combination of contrast energy and the grouping constraint of its neighboring candidates:

(11) $$W\left(B_{i}\right)=P_{in-curv}\left(B_{i}\right)+\sum_{B_{j}\in N\left(B_{i},r_{g}\right)}P_{cont}\left(B_{i},B_{j}\right)\cdot P_{sim}\left(B_{i},B_{j}\right)\cdot E\left(B_{j}\right)$$

where $N(B_{i},r_{g})$ denotes a set of neighborhood contour candidates that partially or totally fall on the discs of radius $r_{g}$ centered at the terminal points of $B_{i}$. $r_{g}$ should be set as a small value to establish the good continuity of the neighbor candidates adjacent to $B_{i}$. In this work, we set $r_{g}=5$ pixels.

Then, the final output of each pixel of the image is the combination of the weights of each candidate computed from hierarchical cues over all scales:

(12) $$W=\sum_{j=0}^{s}\sum_{i}\beta_{\sigma_{j}}\mathrm{IP}\left(W\left(b_{i},\sigma_{j}\right)\right)$$

where $\beta_{\sigma_{j}}$ indicates the relative contribution of each scale and IP(·) denotes the inverse Gaussian pyramids processing to transform the contour candidates at $\sigma_{j}$ to the original scale $\sigma_{0}$.

## 4. Tests and Results

In this section, we evaluate the performance of our model on a popular Berkeley Segmentation Dataset (BSD300 and BSD500) provided by Malik *et al*. [1,26]. This dataset has been frequently used as a benchmark of contour detection algorithms. Each image in the dataset was labeled by 5∼10 human subjects. We first evaluated our superpixel-based contour candidate generation method; then, we further tested the performance of our algorithm and compared it to other state-of-the-art algorithms.

To compare the algorithms conveniently, we took the sum of weights of contour candidates over each pixel as the probability of being a true contour after an operation of non-maxima suppression [1,26]. We also computed the so-called F-measure [26],

(13) $$F=\frac{2PR}{P+R}$$

where *P* and *R* represent the precision and recall, respectively, which have been widely used to evaluate the performance of edge detectors [26]. Precision reflects the probability that the detected edge is valid, and recall denotes the probability that the ground truth edge is detected.

### 4.1. Evaluation of the Superpixel-Based Contour Candidate

**Figure 4:**
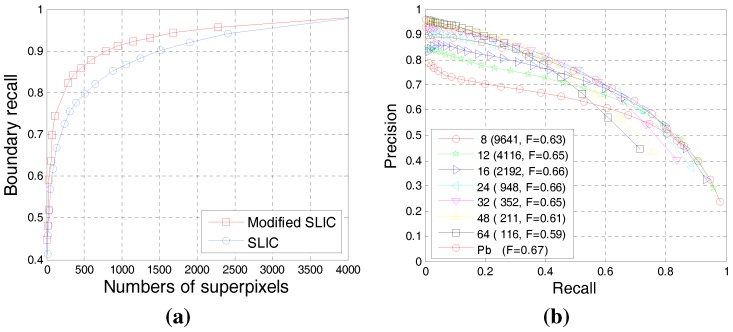
(**a**) Plot of the boundary recall w.r.t. the number of superpixels; (**b**) P-Rcurve comparison with our method under different initial grid intervals and *Pb* [26] ; values inside those parentheses of the legend are the number of superpixels corresponding to the initial grid interval and its F-scores.

A basic assumption of our method is that superpixel boundaries adhere well to the contours of meaningful objects. If this assumption is valid, then the superpixel boundaries can be taken as contour candidates. Boundary recall is a standard measure of boundary adherence [66], and this parameter measures the fraction of the ground truth edges that falls within at least *m* pixels of a superpixel boundary (m=2 according to [66]). A high boundary recall indicates that very few true edges are missed. Experiments in [66] had discussed the boundary recall changes of original SLIC with increasing numbers of superpixels. We further evaluated our modified SLIC processing on the BSDS dataset [1]. Figure 4a shows the boundary recall with different numbers of superpixels generated by the original SLIC and our modified approach. We can see from Figure 4a that the boundary recall increased with the number of superpixels. Our modified SLIC demonstrated a better performance than the original SLIC. When the number of superpixels reached 4000, the boundary recall was 0.98, which experimentally verified the completion of our candidate generation method. Note that the boundary recall already reached 0.96 when the number of superpixels is 2000. That is, the doubled number of superpixels only brings a 0.02 increase of boundary recall. To ensure subsequent calculation efficiency without evidently reducing the boundary recall, we set the number of superpixels as 2000. Correspondingly, we perform SLIC with four initial centers of different offsets ( (0,0), (0,S/2), (S/2,0), (S/2,S/2) ) relative to the initial centers of the original method and set the number of approximately equally-sized superpixels as k=500 for each initial center (the initial grid interval *S* is 16 for approximation correspondingly).

We further evaluated the performance of our superpixel-based candidate generation method on BSDS500. Here, we only use low-level cues (luminance and color) to weigh the candidates at the original scale. Figure 4b illustrates a comparison based on the precision-recall (PR)curve with our method under different initial grid intervals and the previously proposed method *Pb* [26]. When the initial grid interval *S* was set as 16, our method yielded good results (F=0.66), close to *Pb* [26] (F=0.67), which uses texture, as well as luminance and color and requires a training process. In addition, the method with the small initial interval showed better performance in the part with high recall, while that with a large initial interval exhibited better performance in the part with high precision. Therefore, we can use multiscale strategies to improve the effectiveness of our algorithm.

### 4.2. Experimental Evaluation of Our BICW Algorithm

**Figure 5:**
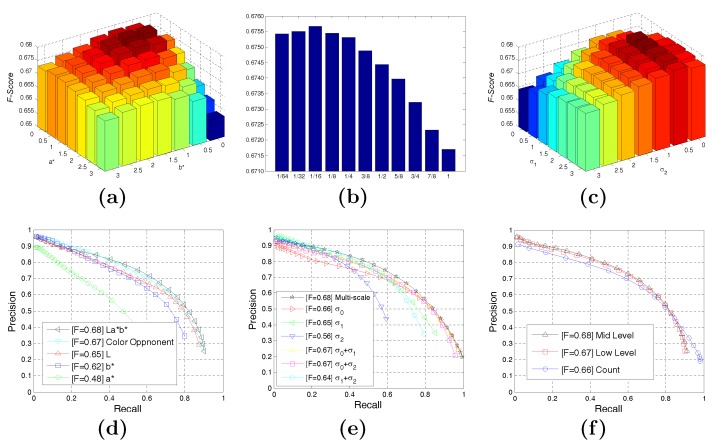
Performance tests with the parameters in our model. (**a**) Performance with the changing of low-level cue parameters; (**b**) performance with the changing of mid-level cue parameters; (**c**) performance with the changing of the relative contribution of each scale; (**d**) P-R curves of $L^{*}a^{*}b^{*}$-based and color opponent-based methods; (**e**) P-R curves of each scale; (**f**) P-R curves of the performance of each part in our algorithm.

The parameters involved in our model are summarized in Table 1. The parameters mainly come from three aspects: low-level cue computation, mid-level cue computation and multi-scale combination. We experimentally analyzed these parameters. To ensure the integrity of the evaluation, we only employed the training set (200 images) of the BSDS500 dataset [1] for parameter setting during the optimization phase and then benchmarked our method on the test set (200 images). We use the expectation maximization (EM) algorithm to tune these parameters. Our algorithm achieves an optimal performance (F=0.68 on the training set of BSDS500 [1]) with the settings shown in Table 1. Figure 5 provides a performance measure with the changing of parameters in our model.

**Table 1:** Parameters involved in our model.

Parameter	Description	Equation	Setting
$\alpha_{L},\alpha_{a},\alpha_{b}$	Low-level: Relative contribution of each color channel	(6)	1,0.5,1
δ	Mid-level: Sensitivity of connection probability with contrast	(9)	1/16
$\beta_{\sigma_{0}},\beta_{\sigma_{1}},\beta_{\sigma_{2}}$	Scales: Relative contribution of each scale	(12)	1,1,0.5

For the parameters of low-level computation, as shown in Figure 5a, we set $\alpha_{L}=1$ for normalization and discuss the performance with the changing of $\alpha_{a}$ (seven values within [0 3]) and $\alpha_{b}$ (seven values within [0 3]). The corresponding parameters of the optimal performance were $\alpha_{a}=0.5$ and $\alpha_{b}=1$. Besides $\alpha_{a}=0$, the F-scores decrease with the increasing of $\alpha_{a}$ at the same $\alpha_{b}$. The contribution of each color channel is also shown in Figure 5d. The channel $a^{*}$ exhibited the poorest performance, so that the the contribution of $a^{*}$ was smaller than that of other channels. The information from channel $a^{*}$ is often missing, which leads to a sharp drop of the recall. Therefore, the relative contribution of channel $a^{*}$ is reasonably smaller than that of other channels. We also compared our LAB-based luminance and chromatic information extraction method with the color-opponent-based method proposed by [74]. We extracted the red-green (R-G), red-cyan (R-C), yellow-blue (B-Y) and white-black (Wh-Bl) color opponent channels instead of lab channels in the computation of low-level cues and perform the subsequent computations. The achieved performance was similar to the ones computed with LAB channels with marginal color values for $a^{*}$ and $b^{*}$. This is mainly because CIELAB is also a kind of color-opponent space with dimension $L^{*}$ for lightness (luminance) and $a^{*}$ and $b^{*}$ for the color-opponent dimensions. In this work, we use CIELAB for simplification.

For the parameters of mid-level cues, the corresponding parameter of the optimal performance was δ=1/16, as shown in Figure 5b. For the parameters of scales, $\beta_{\sigma_{0}}=1$ was set to achieve normalization and to discuss the performance with the changes in $\beta_{\sigma_{1}}$ (seven values within [0 3]) and $\beta_{\sigma_{2}}$ (seven values within [0 3]). When $\beta_{\sigma_{1}}=1$ and $\beta_{\sigma_{2}}=0.5$, we get an optimal performance. We also examined the performance of each scale and combination of each scale, whose P-R curves were shown in Figure 5e. Most of the information was retained at the smallest scale of $\delta_{0}$, and the details were mostly preserved, so the performance of $\delta_{0}$ is better for the measure of recall. Since only the main contours were retained at the scale $\delta_{2}$ ($4\delta_{0}$), the recall of this scale drops sharply. Scale $\delta_{1}$ performs better for precision, as both details and main contours are retained well. We used the weighted sum approach to aggregate the results of different scales. With this method, the main outlines emerging at various scales are applied to generate higher impact, leading to higher performance than that of the single scale.

The performance of each part in our algorithm is shown in Figure 5f. The blue curve in Figure 5f shows the performance of our candidate-based contour detection framework without weighting by visual cues. The weights are just the occurrence of pixels in the contour candidate set. It actually represents the performance of our modified superpixel-based contour candidate generation method. The red and black curve indicate the performance of our algorithm weighted by low-level and mid-level visual cues.

**Figure 6:**
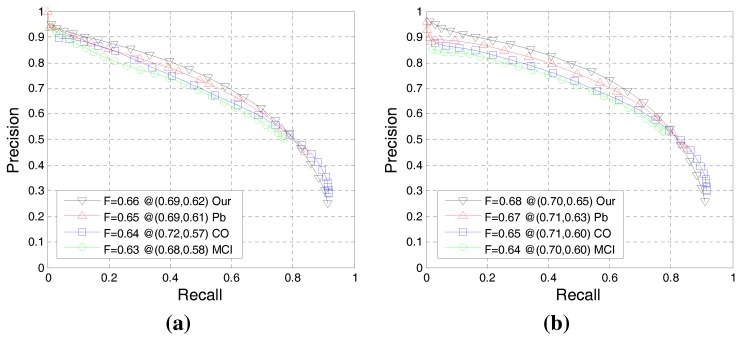
(**a**) The overall performance of the P-R curve on BSDS300 [26]; (**b**) the overall performance of the P-R curve on BSDS500 [1].

**Figure 7:**
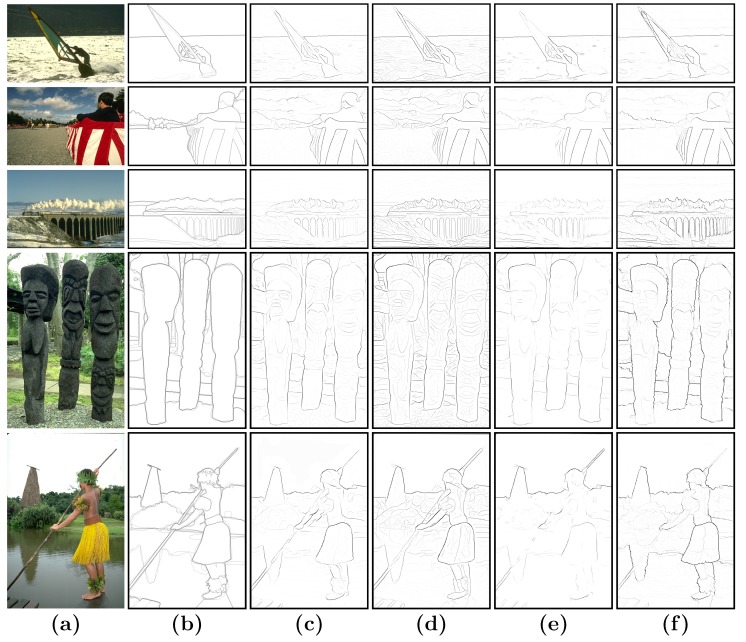
(**a**) Image; (**b**) human; (**c**) *Pb* [26]; (**d**) *CO* [48]; (**e**) *MCI* [55]; (**f**) ours.

In the following step, these parameter settings were fixed in different scenes when the proposed method is compared to other models. Figure 6 illustrates the P-R curve-based comparison of our BICW with the typical learning method *Pb* [26] and two state-of-the-art biologically-inspired methods, *CO* [55] and *MCI* [48], on the datasets of BSDS300 [26] and BSDS500 [1]. Figure 6 reveals that the proposed model outperforms the *Pb* [26] method, but employs less information (it does not use texture information). In addition, our model also outperforms *CO* [55] and *MCI* [48], which are state-of-the-art biologically-inspired models proposed within the last three years. Figure 7 presents qualitative comparisons on some images.

Table 2 lists further comparisons with other state-of-the-art biologically-inspired or non-learning methods on BSDS300 [26] and BSDS500 [1]. The state-of-the-art algorithm *gPb* [1] achieved a higher F-measure (F = 0.70) than our model. This method is particularly time consuming because of the complex texture calculations and globalization with spectral clustering. In the *gPb* [1] algorithm, the computation is conducted by placing a circular disc in each pixel split into two half-discs with a diameter at a certain angle. The measure of the boundary strength is computed in each pixel (more than 150,000 pixels) with eight orientations. In our method, the contour candidates are reduced to 30,000. The mean computation time to compute one contour map with *gPb* [1] is 191 s, while our algorithms only takes 13 s (the computer used here is Intel Core 2, 2.5 GHZ with 8.0 G RAM). Our method is a littler slower than *CO* [48] and *SCO* [75]. Note that, *CO* [48] and *SCO* [75] only perform at a single scale, because their extension into multi-scale space did not yet produce clear performance improvements [48,75]. The computation time of our method is promising as a multi-scale method. Therefore, this result indicated that our model yields a good trade-off between performance and complexity. In addition, our proposed method is very suitable for parallel computing, which is a future direction to optimize our model.

Another point worth noticing is that [76] pointed out that the BSDS benchmark [1,26] has potential pitfalls. Psychophysical experiments in a previous work [76] showed that several “weak” boundary labels are unreliable and may contaminate the benchmark. They pointed out that the current benchmarking protocol encourages an algorithm to bias towards those problematic “weak” boundary labels. Although a consensus to define a new standard has yet to be obtained, new directions have been proposed to focus on the new problem of strong boundary detection. These boundaries are more likely to be meaningful contours. For example, as shown in Figure 3, cluttered edges of the ornamentson the legs were also labeled as boundaries by specific subjects. These “weak” boundaries are suppressed by our method. Figure 7 gives several typical examples, which clearly demonstrate that our algorithm is consistent with the strong boundaries of the consensus labels.

**Table 2:** Quantitative comparison of various models on the BSDS300 [26] and BSDS500 [1] images with the F-score.

Methods	F-Score on BSDS300	F-Score on BSDS500	Time (s)
*Pb* (2004) [26]	0.65	0.67	51.00
*gPb* (2012) [1]	0.70	0.71	191.15
*CO* (2013) [48]	0.64	0.65	6.51
*MCI* (2014) [55]	0.62	0.64	48.60
*SCO* (2015) [75]	0.66	0.67	7.68
Ours	0.67	0.68	13.13

## 5. Conclusions

In this study, a BICW framework that uses superpixel-based candidates and hierarchical visual cues is proposed to detect meaningful contours. In contrast to previous models with pixel-based contour detection, a modified superpixel generation processing has been introduced to generate a contour candidate set. The candidates are then weighted by integrating the information from multiple biologically-inspired cues. We extract the low-level visual local cues on the basis of the contrast energy to weigh the contour intrinsic property. Gestalt principle-based mid-level visual cues, namely curvature consistency, continuation and similarity, are integrated to weigh the contour grouping constraint. The final output is generated by considering information at multiple scales.

The main contribution of the work could be summarized as follows. (1) A candidate weighting framework is designed for the task of contour detection. To our knowledge, this is the first attempt to build a contour candidate set of a line segment rather than a candidate set of pixels using the superpixel-based method. Other visual features could be easily integrated into this unified framework in the future; (2) A model based on biologically-inspired cues is designed to weigh the candidates. Our model is distinguished mainly by how to use and integrate the multiple visual cues for weighing the candidates; (3) The final output is generated by integrating the information at multiple scales. This strategy employs the property that salient and meaningful contours are more likely to be retained at different scales. The weights of the meaningful contours are boosted when they are integrated from multiple scales; (4) The result shows that our model achieves a good trade-off between performance and efficiency. The method is also very suitable for parallel computing, which is a future direction for optimization.

To summarize, we propose a biologically-inspired framework for contour detection using superpixel-based candidates and hierarchical visual cues. Experimental results based on the BSDS benchmark show that the proposed framework exhibits promising performances in terms of capturing meaningful contours in complex scenes.
